# Prediction of Feed Efficiency and Performance-Based Traits in Fish via Integration of Multiple Omics and Clinical Covariates

**DOI:** 10.3390/biology12081135

**Published:** 2023-08-15

**Authors:** Tim Young, Olivier Laroche, Seumas P. Walker, Matthew R. Miller, Paula Casanovas, Konstanze Steiner, Noah Esmaeili, Ruixiang Zhao, John P. Bowman, Richard Wilson, Andrew Bridle, Chris G. Carter, Barbara F. Nowak, Andrea C. Alfaro, Jane E. Symonds

**Affiliations:** 1 Aquaculture Biotechnology Research Group, Department of Environmental Science, School of Science, Private Bag 92006, Auckland 1142, New Zealand; 2 The Centre for Biomedical and Chemical Sciences, School of Science, Auckland University of Technology, Private Bag 92006, Auckland 1142, New Zealand; 3 Cawthron Institute, Nelson 7010, New Zealand; 4 Institute for Marine and Antarctic Studies, University of Tasmania, Hobart Private Bag 49, Hobart 7005, Australia; 5 Tasmanian Institute of Agricultural Research, University of Tasmania, Hobart 7005, Australia; 6 Central Science Laboratory, Research Division, University of Tasmania, Hobart 7001, Australia; 7 Blue Economy Cooperative Research Centre, Launceston 7250, Australia

**Keywords:** integrated multi-omics, metabolomics, proteomics, microbiomics, machine learning, random forest, feed efficiency, aquaculture

## Abstract

**Simple Summary:**

Aquaculture plays a key role in many emerging economies. Sources of fish for human consumption now exceed those from capture fisheries. However, the high cost of food to feed fish limits investment returns. Physiological inefficiencies in the way fish digest and assimilate nutrients and energy from food can be improved to lower the amount of feed required to farm fish whilst reducing waste and enhancing environmental sustainability. Being able to measure this ‘feed efficiency’ is crucial to develop strategies to improve it, yet we do not currently have a good means of doing this. With a focus on salmon, this research uses detailed physiological information involved in fish metabolic processes to better understand the mechanisms that can contribute towards better feed efficiency and growth, and at the same time enable development of models to accurately predict such measures. Our findings can be used to improve the efficiency of the salmon aquaculture industry and reduce the impact of farming practices on the environment.

**Abstract:**

Fish aquaculture is a rapidly expanding global industry, set to support growing demands for sources of marine protein. Enhancing feed efficiency (FE) in farmed fish is required to reduce production costs and improve sector sustainability. Recognising that organisms are complex systems whose emerging phenotypes are the product of multiple interacting molecular processes, systems-based approaches are expected to deliver new biological insights into FE and growth performance. Here, we establish 14 diverse layers of multi-omics and clinical covariates to assess their capacities to predict FE and associated performance traits in a fish model (*Oncorhynchus tshawytscha*) and uncover the influential variables. Inter-omic relatedness between the different layers revealed several significant concordances, particularly between datasets originating from similar material/tissue and between blood indicators and some of the proteomic (liver), metabolomic (liver), and microbiomic layers. Single- and multi-layer random forest (RF) regression models showed that integration of all data layers provide greater FE prediction power than any single-layer model alone. Although FE was among the most challenging of the traits we attempted to predict, the mean accuracy of 40 different FE models in terms of root-mean square errors normalized to percentage was 30.4%, supporting RF as a feature selection tool and approach for complex trait prediction. Major contributions to the integrated FE models were derived from layers of proteomic and metabolomic data, with substantial influence also provided by the lipid composition layer. A correlation matrix of the top 27 variables in the models highlighted FE trait-associations with faecal bacteria (*Serratia* spp.), palmitic and nervonic acid moieties in whole body lipids, levels of free glycerol in muscle, and N-acetylglutamic acid content in liver. In summary, we identified subsets of molecular characteristics for the assessment of commercially relevant performance-based metrics in farmed Chinook salmon.

## 1. Introduction

Meeting global demands for sources of sustainable food protein is one of our greatest challenges. Aquaculture is uniquely poised to help resolve this for future populations. Creation of a thriving and viable ‘blue economy’ will greatly expand resource availability, capacity, and opportunity. However, increased seafood production will need to be complemented by reductions in environmental impact and improvements in resource efficiency [1,2]. Fish nutrition and feed development are key areas of focus to support sector advancement and drive aquaculture growth.

Feed efficiency (FE) is the efficiency with which food is utilised by animals for productive purposes such as growth. Commonly expressed as the Feed Conversion Ratio (FCR), this measure is the ratio between feed intake and body weight gain over a particular period. FE is the inverse of FCR, with lower FCR values indicating higher (enhanced) FE; from herein the term FE is primarily used in discussion for simplicity. In salmonid aquaculture, feed typically comprises 50–60% of total production costs, and excess provisions through uneaten and/or poorly digested food can cause adverse environmental impact [3,4]. FE is thus a highly important consideration for farming fish, and trait enhancement can improve sector profitability and sustainability. Yet, FE trait improvement is often overlooked by selective breeding programmes because it is difficult to measure in individual fish and the genetic underpinnings are not well understood [5,6].

FE is a complex trait which is influenced by numerous inextricably linked physiological processes, including social behaviour [7], feed intake [8], digestion capacity [9,10], nutrient absorption and assimilation [11,12], metabolism [13], and genetics [5,14,15]; processes which are further governed by a myriad of environmental factors [16,17]. Feed efficient fish tend to eat smaller meals whilst maintaining higher growth rates than inefficient conspecifics. We recently demonstrated in farmed Chinook salmon (*Oncorhynchus tshawytscha*) that fish displaying higher FE also have higher capacities for protein, lipid, and energy retention, and lower minimal routine metabolic rates [18]. These findings reveal that salmon with higher FE are also more efficient in their energy expenditure, requiring less energy for maintenance processes and presumably allowing more energy to be allocated to growth.

Providing further mechanistic insight into FE in farmed salmon, we have applied exploratory metabolomic- and proteomic-based approaches to screen for metabolite and protein biomarkers associated with divergent FE phenotypes. Using secondary bioinformatics techniques to reveal functional biochemical pathway information, findings highlighted that fish with high FE display greater capacity for protein synthesis in white muscle tissue, intestine, and liver, and increased energy production from elevated rates of lipid metabolism in the liver and brain [19,20,21]. Conversely, salmon with low FE displayed signatures indicative of protein degradation process (proteolysis and amino acid catabolism) being prominent in muscle, liver, and the intestine [21,22].

While these outcomes inform some physiological processes coupled to FE, additional information is believed obtainable through incorporation of broader biological features with opportunity to develop FE trait prediction capability. To this end, we have subsequently assessed a range of samples in Chinook salmon displaying variable FE phenotypes, including clinical covariates (i.e., multiple health indices, haematology parameters, and known diagnostic blood biomarkers), crude chemical composition data, lipid composition, multi-sample proteomics, dual-technique metabolomics, and faecal microbiomics. Associations between FE and clinical biomarkers, the digestive microbiome, and body composition in salmon are currently unknown. Integration of these data form the basis of the current study.

Integration of multi-omics data is challenging due to their heterogeneity, yet is considered essential to decipher complex mechanisms underpinning phenotypic variations and gain holistic biological understanding [23]. Strategies to investigate cross-omic associations are developing rapidly and a wide array of methods are available [23,24,25,26,27]. Various machine learning principals and/or analyses of network architecture are typically applied under one of two primary frameworks—data-driven or knowledge-based integration—with method selection being guided by characteristics of the data at hand.

Knowledge-based approaches combine omics data from a functional perspective via pathway-based modelling [25,26,27]. Leveraging the collective wealth of genomic and biochemical knowledge in the public domain, genome-scale metabolic models and networks can be interrogated to link experimental data and extract unique information. Such approaches are suited to studies involving homogeneous sample types and sets of omics data with known functional relationships (e.g., transcriptomic—proteomic—metabolomic). Data-driven approaches on the other hand typically rely on machine learning algorithms to select relevant features from multi-omics datasets for interpretative and/or predictive purposes without a priori assumptions [23,28,29].

Our current data are particularly well-suited to a data-driven integration strategy due to collection of different sample types, inclusion of non-omics-based data, use of a high number of diverse datasets, and a lack of known biological associations among them. Multi-omics and clinical covariate data have different modalities and are by their nature meta-dimensional. These different data subsets, referred from now as ‘layers’, may each contribute information valuable for providing insight into, or predicting, phenotypic traits. However, challenges in the development of data-driven integration strategies are associated with overlapping predictive information within individual layers, differences in levels of predictive information between layers (which may also be dependent on the phenotypic traits under study), and requisite to consider existing interactions between variables across data layers [29]. To overcome these issues, Hornung and Wright [29] recently developed a prediction modelling approach for combining multi-omics and clinical covariates called ‘Random Block’; a variant of the random forest (RF) decision tree algorithm. 

RF is an ensemble-based machine learning method able to capture complex dependency patterns between covariates and phenotypic traits [29]. RF is widely applicable to classification and regression problems, is robust to outliers, easily handles heterogenous data types (normalisation not required), is generally resilient to overfitting, does not rely on data distribution assumptions, can discern non-linear relationships, and computes feature importances in model predictions [30,31]. However, early use of RF in multi-omics integration relied on concatenated single matrix datasets and suffered from problems associated with unequal layer representation. The new Random Block variant addresses this integration problem by keeping the data layers separate and randomising the layer choice for each node split. When succession of the layers is randomised, predictions of the individual trees differ more widely. Furthermore, since predictive information contained within each layer overlap, the omission of particular layers at each split has a limited negative impact on the forecasting ability of each tree. Block-based RF has shown to out-perform numerous alternative data-driven multi-omics integration strategies for prediction modelling of phenotypic traits [29,32,33,34]. The algorithm’s versatility allows both the importance of individual features and the contributions of entire layers towards prediction accuracy to be assessed.

Herein, we utilise the powerful Random Block regression-based method to integrate 14 layers of covariate information obtained from the analysis of matched samples from individual fish displaying a continuum of FE. Our primary aim was to explore utility of leveraging multiple omics and clinical covariates for the prediction of FE phenotypes in farmed salmon. We further sought to extend application of this rich meta-dimensional data for the prediction of other performance-based traits related to feed intake and growth. Our specific objectives were to: (1) establish associations among the different data modalities, (2) construct a suite of trait prediction models and benchmark their accuracies, (3) evaluate relative contributions each information layer provides for predicting the different traits, and (4) gain new insights into mechanisms underpinning feed efficiency.

## 2. Materials and Methods

### 2.1. Experimental Design

Fish and their corresponding layers of multiple omics and clinical information were derived from a larger trial designed to establish the effects of water temperature and dietary ration on various performance metrics, such as feed efficiency (FE) and growth; see ‘Supplementary Methods S1’ for detailed descriptions of the fish, experimental design, and rearing conditions. Briefly, an all-female cohort of juvenile Chinook salmon were obtained from a commercial hatchery (Mt Cook Alpine Salmon; Canterbury, New Zealand), individually tagged, and reared for eight months at the Cawthron Aquaculture Park Finfish Research Centre (Nelson, New Zealand) in nine 8000 L tanks (176–187 fish per tank) on a freshwater recirculating aquaculture system at 17 °C. To promote FE variation, tanks were assigned one of three feed rations (60%, 80%, and 100% (satiation)), with three replicate tanks per ration; the 60 and 80% rations were calculated daily based on the satiation ration adjusted for biomass differences.

**Figure 1:**
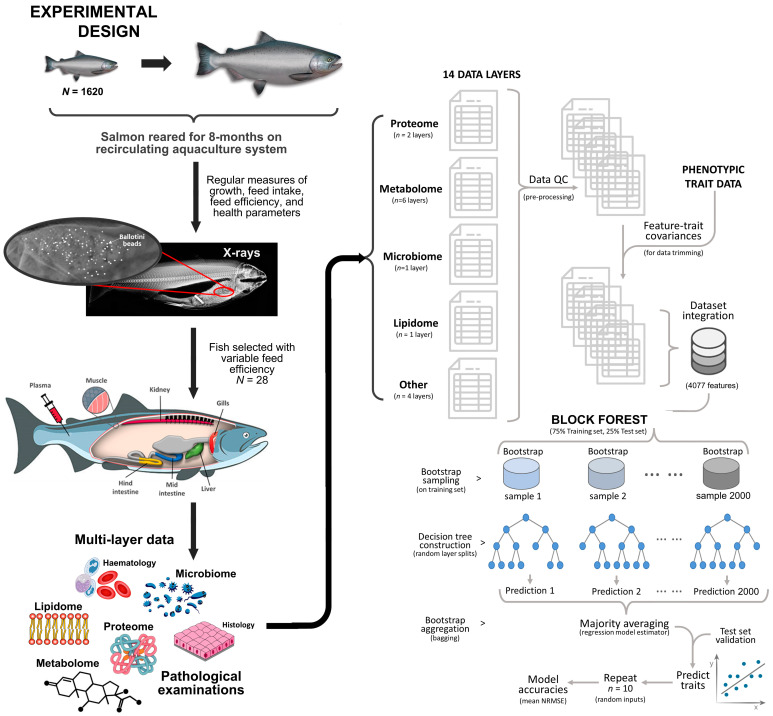
Schematic overview of the experimental design and data analysis workflow.

During the experimental period, fish were assessed four times and individual FE was estimated in three sequential phases. On completion of the trial, salmon from each tank were selected based on their overall health status and consistencies of their FE trait ranks across the FE assessments. Various samples from these fish were assigned to a broad range of evaluations involving omics (i.e., microbiome, proteome, metabolome, proximate and lipid composition) and/or assessments with clinical relevance (i.e., macroscopic observations, blood chemistry parameters, haematology). We acquired matched information for 28 fish across all samples and layers of multiple omics and clinical covariates (Figure 1). 

### 2.2. Fish Handling and Sampling

Fish were handled in accordance with protocols approved by the Animal Ethics Committee of the Nelson Marlborough Institute of Technology (Ref: AEC2018 CAW01). During phenotypic trait measurements, fish were either sedated with tricaine methanesulfonate (mid-trial assessments) or euthanised first by anaesthetic overdose with AQUI-S^®^ (end-of trial assessments). Immediately following euthanisation, blood samples were withdrawn for haematological and plasma-based biochemical analyses, anatomical health assessments were made, and dissections were performed to obtain liver, muscle, and digesta/faecal samples.

### 2.3. Phenotypic Traits

Performance-based traits were assessed following [19], yielding four measures of growth (i.e., fork length, body weight, condition factor, specific growth rate), three measures of feed intake (i.e., mean daily feed intake (DFI), mean daily feed intake per bodyweight (DFI BW^−1^), the mean proportion of the feed intake (PFI) of an individual fish based on its share of the meal within a tank), and three measures of feed efficiency (i.e., feed conversion ratio (FCR)).

#### 2.3.1. Growth

Fork length (FL (mm)) and body weight (BW (g)) were measured on four occasions during the trial, with Fulton’s K condition factors (CF) being calculated from these values after each assessment:

$$CF=100\times \frac{BW}{{FL}^{3}}$$

End-of-trial FL and BW values were used as dependent variable traits in prediction models, whereas for CF the mean of all four measures were used. Specific growth rates (SGR (% mass per day)) between assessments were calculated as:

$$SGR=\frac{\ln BW\;gain}{N\;Days}\times 100$$

The mean of the three SGR measures were used as model responses. 

#### 2.3.2. Feed Intake

Tank-level feed intake (tFI (g)) was measured daily through quantification of uneaten feed and subtraction from the amount originally provided. Individual feed intake (iFI (g)) assessments were performed using X-radiography, according to [35]. Briefly, on four occasions during the trial, fish were fed customised extruded pellets containing a known concentration of X-ray opaque beads. Fish were X-rayed following these feeding events and numbers of beads within the gastrointestinal tract were counted to provide accurate iFI appraisals for each fish. Mean DFI and mean DFI BW^−1^ were subsequently calculated as:

$$mean\;DFI=\frac{\sum iFI\;measures}{4}$$

$$mean\;DFI\;{BW}^{-1}=\frac{{\sum [iFI}_{\left(i\ldots j\right)}/{BW}_{\left(i\ldots j\right)}]}{4}$$

Measured percentage ‘share of the meal’ (SOM (%)) at each assessment was calculated for each fish from the iFI and tFI values by dividing the individual feed intake by the total feed intake for the tank based on X-radiography; the mean proportion of the feed intake was then calculated as the mean % SOM:

$$\%\;SOM=\frac{iFI}{tFI}\times 100$$

$$mean\;PFI=\frac{\sum \%\;SOMs}{4}$$

#### 2.3.3. Feed Efficiency

Feed conversion ratio (FCR) was used as the measure of individual FE, where FCR is the ratio between the mass of food consumed and the BW gain over a particular period. FCR was calculated using two different methods. The first method (FCR1) used the total food eaten by each tank (accounting for the feed recovered), the mean percent share of the meal of each fish calculated using the daily feed intake, and the total weight gain of the fish between assessment periods.

First, we calculated the percent share of the meal for each fish (% SOM) as previously described. The mean percentage of the meal eaten for each fish was then determined based on the assessments at the start and end of each period, for example for Assessment 1 (A1) to Assessment 2 (A2):

$${mean\;\%\;SOM}_{\left(A1\;to\;A2\right)}=\frac{{\%\;SOM}_{\left(A1\right)}+{\%\;SOM}_{\left(A2\right)}}{2}$$

To estimate the total amount of feed eaten by an individual fish from A1 to A2, the total amount of feed consumed by the tank over this period (calculated as the total feed given to the tank minus the total feed recovered (i.e., ∑tFI values A1 to A2)) was multiplied by the mean % SOM for that period, for each individual fish:

$$Total\;feed\;intake={\sum tFI}_{\left(A1\;to\;A2\right)}\times {mean\;\%\;SOM}_{\left(A1\;to\;A2\right)}$$

Individual feed conversion ratio (FCR1) was then calculated as the total feed eaten by an individual (A1 to A2) based on the mean % SOM divided by the total weight gain (g) of the individual (A1 to A2):

$$FCR1=\frac{Total\;feed\;intake}{Total\;weight\;gain}$$

The second method (FCR2) used the mean daily feed intake estimated at the time of the two assessments and the daily weight gain during that period. Individual FCR was calculated as the mean daily feed intake by an individual (for A1 and A2) divided by the daily weight gain of the individual (weight difference between A1 and A2, divided by the number of days between assessments).

$$FCR2=\frac{{mean\;DFI}_{\left(A1\;and\;A2\right)}}{Daily\;weight\;gain}$$

‘Mean FCR1’ and ‘Mean FCR2’ are the means of the three FCR values obtained for each assessment period using the FCR1 and FCR2 methods, respectively. ‘Last FCR1’ and ‘Last FCR2’ are the FCR values for the last (third) measurement period using the FCR1 and FCR2 methods, respectively. These response variables were selected to represent long-term FE phenotypes (i.e., the Mean FCRs) and a snapshot of those reflective of the FE phenotype at the time of sampling for tissues and biofluids (i.e., the Last FCRs).

### 2.4. Model Covariates

Tissues and biofluids were subjected to a range of analyses to generate the model covariates for this study. Across all samples and measurements, a total of 4077 features were retained for inclusion as explanatory variables in the Random Block models (Table 1). Complete lists of all model covariates are provided in Supplementary Data. 

#### 2.4.1. Microbiome Layer

Microbial profiling of faecal samples was performed as described by [36]; refer to ‘Supplementary Methods S2’ for a detailed description. Briefly, bacterial DNA was extracted using a ‘NucleoSpin Soil’ kit according to the manufacturers’ instructions. Polymerase chain reaction was performed using the bacterial universal primers 27F and 519R. To evaluate the bacterial DNA, 2 × 300 bp pair-ended amplicon sequencing of the V1–V3 region of the 16S rRNA gene was performed using a MiSeq Illumina platform.

Data were trimmed, joined, and demultiplexed, then filtered for low quality reads. Taxonomic assignments were made using the ‘Seed 2’ pipeline, according to [37]. Following sequence alignment, denoising, chimera checking, and clustering, operational taxonomic units were optimised and defined then classified against the Silva non-redundant 16S rRNA database (www.arb-silva.de/, accessed on 16 December 2019). Microbial data were pre-processed by filtering out rare amplicon sequence variants (ASVs; those with less than two reads in at least two samples, leaving a total of 135 ASVs) and by transforming their read counts to centred-log ratios following recommendations from [38] when dealing with compositional data.

#### 2.4.2. Proteome Layers

Samples of liver and muscle were processed for proteomic profiling as described by Esmaeili et al. [19]; refer to ‘Supplementary Methods S3’ for a detailed description. Briefly, tissues were homogenised in denaturation buffer and protease inhibitor cocktail. Crude protein was reduced and alkylated via standard techniques, then digested using the ‘SP3’ method according to [39]. Following acidification and de-salting, digests were analysed on a liquid chromatography mass spectrometry (LC-MS) system (LC: Thermo Scientific^TM^ Ultimate 3000 RSLCnano) equipped with a reverse phase (RP) column, nanospray ion source, and a hybrid quadrupole-orbitrap mass spectrometer (MS: Thermo Scientific^TM^ Q-Exactive HF). Proteomic analysis deployed a combination of data-dependent acquisition (DDA) and data-independent acquisition (DIA) mass spectrometry approaches.

Spectral libraries for the tissue peptides were generated by off-line micro fractionation of pooled liver and pooled muscle digests into 16 concatenated fractions using microflow high-pH RP high performance liquid chromatography (HPLC), followed by DDA-MS. Individual samples were then analysed by DIA-MS using the same HPLC gradients and extracted median MS2 ion intensities were used for label-free quantitation of protein abundance between samples. DDA- and DIA-MS raw files were processed using Spectronaut software. Each tissue-specific library was generated using the Pulsar search engine to search DDA MS2 spectra against *Oncorhynchus tshawytscha* protein sequences in the National Center for Biotechnology Information (NCBI) database. Spectronaut protein quantitation pivot reports, including protein description, gene names, and NCBI accession numbers, from the 28 fish were uploaded into Perseus software for further data processing. Quantitative values were log_2_ transformed, and proteins filtered according to the number of valid values (N = 3223 variables). Batch effects in the data were removed via an empirical Bayes framework, the ‘ComBat’ method [40], using MetaboAnalyst v5.0 software [41]. 

#### 2.4.3. Metabolome Layers

Plasma, liver, and muscle samples were extracted and derivatised according to established protocols [42,43], with minor modification; refer to ‘Supplementary Methods S4’ for a detailed description. Briefly, metabolites were co-extracted with internal standards (d4-alanine and ribitol) using methanol as a solvent. Sub-aliquots of each extract were simultaneously derivatised using methyl chloroformate (MCF) and trimethyl silylation (TMS) to target organic acid and carbohydrate functional group chemistries, respectively—thus providing two metabolomics datasets for each sample type (i.e., six metabolomics datasets in total). Derivatised samples were analysed according to [44] on a gas chromatography mass spectrometry (GC-MS) system (GC: Agilent 7890B) equipped with a mid-polarity column, an electron impact ion source, and a quadrupole analyser (MS: Agilent 5977B). Spectral data were deconvoluted using Automated Mass Spectral Deconvolution and Identification System (AMDIS) software [45].

In-house libraries of pure standards and the mass spectral library of the National Institute of Standards and Technology (NIST) were used to identify metabolites where possible; unidentified features were retained as variables within the metabolomics data layers. Pooled quality control normalisation was performed using an RF-based algorithm (Systematic Error Removal using Random Forest [46]) to remove systematic errors associated with post-extraction processes (e.g., batch effects); internal standard-based normalisation was subsequently applied to account for error sources associated with the extractions (e.g., variable metabolite recoveries). Aberrant records were filtered via blank subtraction and manual screening. Lastly, data were adjusted to sample-specific biomass where appropriate, missing values were estimated via the ‘half minimum’ approach, and resultant metabolomics entries (N = 556 variables) were autoscaled.

#### 2.4.4. General Composition Layer

Selected elements, crude chemical composition, and some fatty acid class information in whole-body (WB) homogenates and/or the fillet portion of the fish were measured using established methods [47]; refer to ‘Supplementary Methods S5’ for detailed descriptions. Briefly, levels of calcium and phosphorus were measured in whole-body (WB) homogenates via inductively coupled plasma mass spectrometry (ICP-MS). WB crude chemical composition was assessed using validated procedures of the Association of Official Analytical Chemists.

Briefly, values were obtained for crude protein via total Kjeldahl nitrogen (AOAC 981.10), total fat via acid hydrolysis (AOAC 948.15), moisture via gravimetry at 105 °C (AOAC 950.46), and ash via incineration (AOAC 920.153); total carbohydrate content was estimated by difference (i.e., 100 − % protein − % fat − % moisture − % ash). These wet chemistry data were supplemented with WB proximate composition data estimated using near infrared (NIR) spectroscopy and species-specific NIR models we previously validated [48]. NIR models were also employed to obtain proximate composition and information on selected lipid-based characteristics (e.g., fatty acid classes) in fish fillets, extending the ‘general composition’ data layer (N = 28 variables). 

#### 2.4.5. Lipid Composition Layer

Lipids in WB homogenates were extracted via a cold-solvent-modified Folch extraction [49], and their fatty acid profiles (fatty acid methyl esters [FAMEs]; N = 32 variables) were analysed by gas chromatography mass spectrometry (GC-MS); refer to ‘Supplementary Methods S6’ for a detailed description. Briefly, samples were homogenised in a chloroform/methanol/water solvent mix to partition the lipid component into the organic phase. Following removal and concentration of the organic phase, the crude lipid extracts were methylated using a Gerstel MPS (Multipurpose sampler) via saponification with methanolic sodium hydroxide. FAME samples were analysed according to AOAC official methods 963.22.

#### 2.4.6. Blood Biomarker Layer

A targeted suite of clinically relevant blood biomarkers was profiled according to [50]; refer to ‘Supplementary Methods S7’ for a detailed description. Briefly, snap-frozen plasma samples were sent frozen on dry ice to an accredited commercial laboratory (Gribbles Veterinary (GV); Christchurch, New Zealand) for analysis of blood chemistry indices (N = 24 variables), comprising various proteins, metabolites, ions, and their ratios. Analytes were quantified using automated chemistry analysers with assay kits developed by the manufacturer, or via enzyme-linked immunosorbent assays. 

#### 2.4.7. Haematology Layer

Haematology indices (N = 12 variables) were evaluated according to [51]; refer to ‘Supplementary Methods S8’ for a detailed description. Briefly, haematocrit values were recorded immediately after blood withdrawal. Differential cell counts were obtained using two whole blood smears per fish. Air-dried slides were stained (Leishman) and cells were enumerated under the microscope at the GV laboratory. Haemoglobin content was determined on an automated analyser. 

#### 2.4.8. Health Layer

Selected health-relevant indices (N = 5 variables) were integrated as potential trait predictors, comprising: visceral fat score (VFS) according to [51], faecal appearance score (FAS) according to [52], and somatic indices for the heart, liver, and gonad (i.e., organ weight/BW × 100).

### 2.5. Data Analyses

#### 2.5.1. Concordance Analysis

Pair-wise associations among the different multivariate data modalities were examined via Procrustes analyses and followed by procrustean randomisation tests (PROTEST [53]) to test significance of the goodness-of-fit of correspondence statistics. Procrustes-based concordance analysis essentially computes reduced dimensions (e.g., multidimensional scaling (MDS) configurations) for two multivariate datasets and transforms one of the matrices until it has maximum similarity with the other when superimposed. This is achieved by minimising the sum of the squared residual deviations between points for each observation and the identical observation in the target matrix. Procrustes is widely used for measuring global associations between multi-omics datasets.

Each dataset was standardised and the non-metric dimensional scaling matrices calculated on the Euclidean distance: monoMDS(vegdist(decostand(x = dataset, method = “standardize”), “euclidean”)), followed by PROTEST (scores = “sites”, permutations = how(nperm = 999)m scale = T, symmetric = T) using the ‘vegan’ R package. Briefly, PROTEST assesses the correspondence between matching points of two datasets using least-squares orthogonal mapping and tests for concordance significance using a permutational approach. Results were visualised with a heatmap using the ‘ggplot2’ R package (v3.3.5) [54].

#### 2.5.2. Random Block-Based Prediction of Phenotypes

The random forest variant Random Block differs from its predecessor simply with respect to the selection of the split points in the decision trees which make up the forests. Rather than concatenate the data into a single matrix for integration, Random Block keeps the layers separate as inputs and integrates them during tree construction. Layers were separated by covariate class and by sample type to ensure that overlapping features (e.g., the same protein being detected in muscle and liver) were given simultaneous opportunity for inclusion in the models. At the split points, a block (layer) of data is randomly selected and a subset of features contained within that block is then considered for splitting [29]. Split point selection is subsequently performed as in standard random forest using the sampled subset of features. Randomising succession of the blocks used for splitting promotes the rendering of feature interactions across blocks. 

Prior to Random Block prediction, each dataset was trimmed to keep only the most relevant variables associated with the phenotype of interest using the minimal unbiased variable selection (MUVR) algorithm [55]. MUVR uses machine learning to identify minimal-optimal and all-relevant variable sets for regression and classification problems. The algorithm minimises overfitting and false positives in multivariate analysis and improves predictive performance. The MUVR package (v0.0.973) was implemented in R with the following settings: nRep = 35, nOuter = 8, varRatio = 0.9, method = “RF”, and model = “mid”. 

Trimmed datasets were then used as input (with each dataset representing a block) to the Random Block algorithm (v0.2.4) [29] to create predictive models (block.method = “RandomBlock”, num.trees = 2000, nsets = 300, num.trees.pre = 1500, splitrule = “extratrees”, importance = “impurity”). Prioritisation of particular layers were not applied during tree construction since relationships between different covariates and the responses were unknown a priori.

For each phenotype, ten iterations of the process were executed using 75% of the data to create the models (seeds = 30–39), and the remainder to test their performance. This process allowed us to assess the stability of the models and identify potential issues that could affect the results. Prediction accuracies were evaluated and benchmarked by the mean normalised root-mean square errors (NRMSE), also called a scatter index. The root-mean square error (RMSE) is basically a measure of the differences between predicted and observed values and normalising the RMSE enables comparison between datasets of different scales. The lower the NRMSE value, the lesser the deviation of the predicted values from the true response. Here, the RMSE was normalised by the mean and transformed to a percentage following the formula below, where y represents observed values:

$$NRMSE=\frac{RMSE}{\bar{y}}\times 100$$

The mean contribution of each layer (block) for phenotype prediction (based on block/layer selection probabilities) was visualised with stacked bar plots using the ‘ggplot2’ R package. To evaluate the general performance of models based on the integration of all data versus individual datasets, Random Block models were also created per dataset and the performance of each ranked per phenotype and summed across all traits. Results were then displayed as bar plots using the ‘ggplot2’ R package.

#### 2.5.3. Network Analysis and Inter-Layer Associations

Focusing specifically now on FE as the trait of interest, variables selected by the best models for the FE predictions were examined and compared between FCR methodologies (i.e., mean and last FCR1 and FCR2) using an UpSet figure and the ‘UpSetR’ (v1.4.0) R package [56]. Variables consistently found to be important in predicting the FE sub-phenotypes were further investigated by creating a correlation matrix and a co-occurrence network. First, all variables of interest, including the FCR phenotypes, were merged into a single dataframe and normalised. Secondly, Pearson correlations were performed between each variable using the rcorr function of the ‘Hmisc’ (v4.5.0) R package [57]; a heatmap matrix of these values were plotted. Finally, highly significant relationships (*p*-value ≤ 0.001) were displayed in a co-occurrence network using the ‘ggraph’ (v2.0.5) R package [58].

## 3. Results and Discussion

### 3.1. Phenotypic Traits

**Figure 2:**
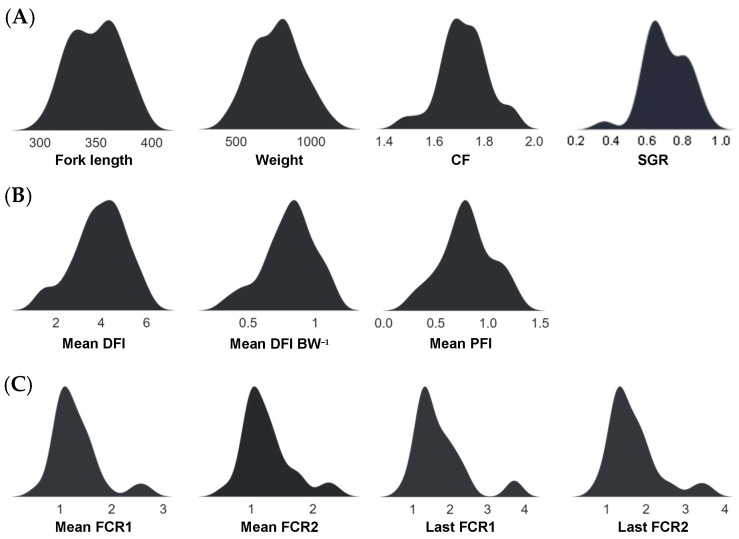
Performance-based trait distributions of salmon (*O. tshawytscha*) used as response variables in the Random Block models, comprising measures of growth (**A**), feed intake (**B**), and feed efficiency (**C**).

The fish phenotypic traits used in this study as Random Block model response variables are displayed as density plots in Figure 2, highlighting their distributions. Traits were generally unimodal, although a relatively small proportion of fish had poor FE values, which were also associated with lower SGR and CF values.

### 3.2. Covariate Layer Associations

To investigate inherent cross-layer association, an initial survey of the multi-omic and clinical covariate data was performed without considering the phenotypic traits of fish. Global covariate associations between the different layers were assessed using Procrustes analysis, which aims to find the best possible correspondence or correlation between two sets of multivariate matrices. Procrustes analysis uses ordination methods to compare high-dimensional datasets by optimally aligning their lower-dimensional projections. Dissimilarity between configurations is iteratively minimised based on a distance metric and the sum of square differences between corresponding points.

**Figure 3:**
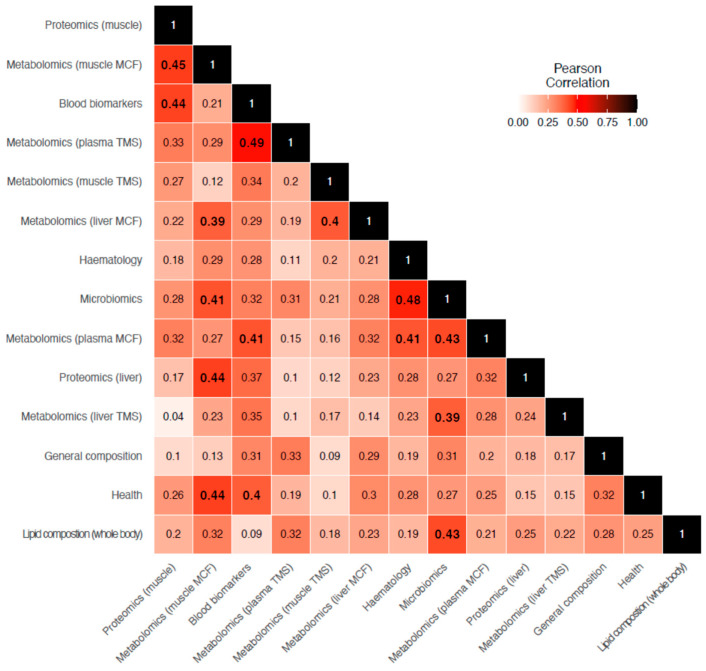
Heatmap of Pearson correlation between datasets derived from PROTEST results. Significant (*p* < 0.05) associations are highlighted in bold.

Significant concordance between many of the layers were detected (Figure 3; bold values only). Highest concordances were observed between layers of similar omics approach (i.e., metabolomics plasma TMS vs. metabolomics muscle MCF data (*r* = 0.49)) and between layers originating from the same sample (i.e., metabolomic plasma TMS vs. metabolomic plasma MCF [*r* = 0.43] and metabolomics muscle MCF vs. muscle proteomics), suggesting associations between these different levels of biological organisation within sample types. In addition, strong and significant correlation between blood biomarkers and health (*r* = 0.44), and between the former and a few of the omics datasets (i.e., proteomics liver (*r* = 0.44), metabolomics liver MCF (*r* = 0.39), and microbiomics (*r* = 0.41)) were observed.

Our study highlights the utility of concordance-based analysis to examine relationships between multi-omics datasets, extending its use beyond some existing microbiome–metabolome exemplars in human models [59,60,61]. Omics-based investigations of fish health is a rapidly evolving area of research important for the development of strategies to confer disease resistance and produce therapeutic interventions [62,63,64,65]. Unravelling cross-omic associations which underly various health conditions in fish is a challenging but crucial task for mapping molecular networks. 

### 3.3. Integrated Random Block Prediction Accuracies

**Figure 4:**
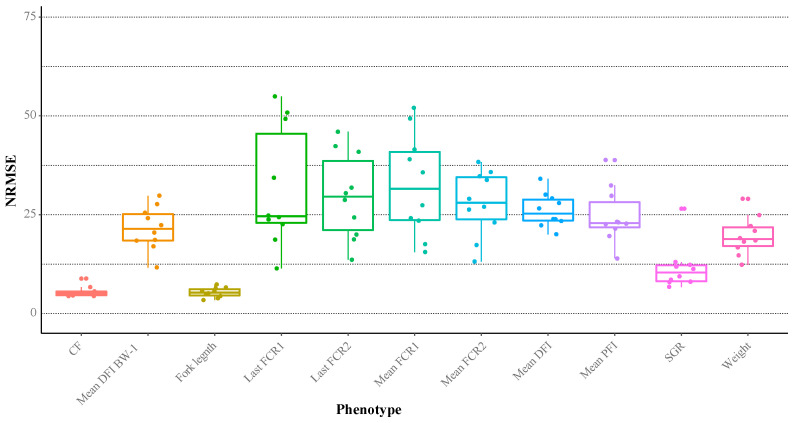
Boxplots of the normalised root-mean square error (NRMSE) in the Random Block phenotype prediction models based on all layers. Data points correspond to the result of each model iteration.

Using the integrated multiple omics and clinical covariates, a suite of Random Block trait prediction models was constructed to benchmark their performance accuracies and reveal scope for potential biomarker identification and interpretation (Figure 4). The most predictable phenotypes included fork length, condition factor and SGR with average mean normalised root mean squared errors (NRMSE) of 5.3%, 5.5%, and 11.5%, respectively. Although practical utilities of these growth models are considered limited since the traits themselves are easy to measure directly, the identities of important covariates within the models may expose underpinning mechanistic foundations. FE measures (our primary trait of interest) were among the most difficult to predict with models showing moderate–high variance in performance (NRMSE’s 11.4–54.9%). However, 23 out of 40 of these model iterations had NRMSE’s < 30%, which provides an encouraging outlook for discovering putative covariate–trait associations.

### 3.4. Covariate Layer Contributions

**Figure 5:**
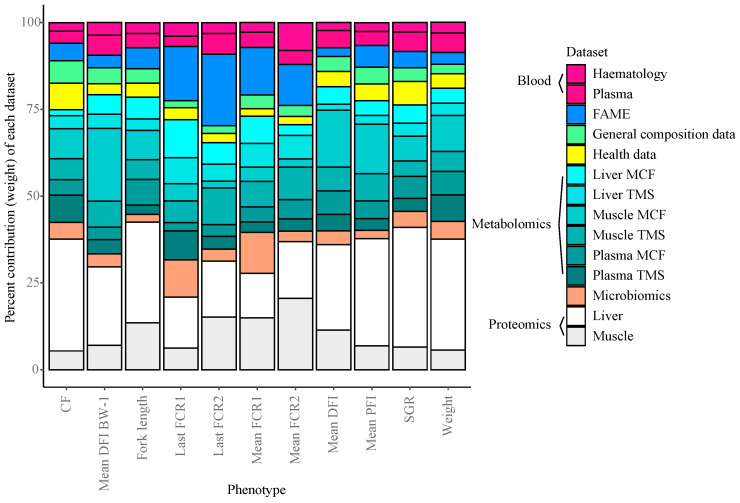
Mean dataset contribution (%) of the Random Block models per phenotype.

During construction of the integrated Random Block models, specific selection probabilities are calculated for each of the different layers. Using these values, the average relative contribution each information layer provides for predicting the phenotypic traits in the Random Block models were assessed (Figure 5). Overall, the datasets which contributed most to trait prediction were the liver and muscle proteomics data, as well as the muscle and plasma MCF and TMS metabolomics data. This aligns with the importance of metabolic processes underlying growth performance variation. Whole body lipid composition (fatty acid (FAME) data) played a greater role in predicting FE phenotypes compared to other performance metrics in general, and faecal microbial composition played a larger role in model performances in two of the four FE measures. Associations between fatty acid profiles and FE have previously been found in cattle [66], yet such relationships in fish are currently unknown [19]. 

**Figure 6:**
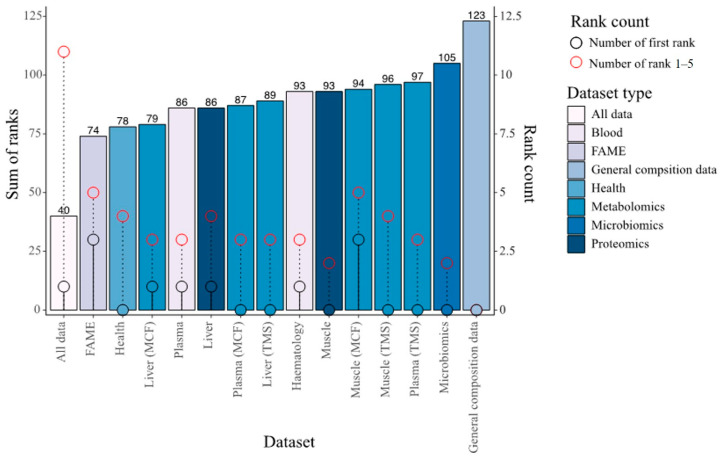
Overall performance (by ranking of NMRSE values per phenotype) of Random Block models trained per dataset. The dataset with the lowest NMRSE per phenotype was given a rank of one while the dataset with highest NMRSE was given a rank of 15 (Random Block models were trained on the 15 different datasets in the figure). The barplot represent the sum of the ranks allocated per phenotype per dataset. The black lollipops indicate the number of times models from the dataset ranked first while the red lollipops show the number of times they ranked within the five best datasets.

Global performance of the integrated datasets with combined modalities versus individual dataset in predicting phenotypes was assessed by ranking the NRMSE results per dataset per phenotype and by summing these ranks (Figure 6). The integrated data and the general composition layer had by far the lowest (N = 40) and highest sum of ranks (N = 123), respectively, while differences between the other datasets were more subtle. Interestingly, the models from the integrated data rank first only once but were consistently among the top five performers. In a relatively similar study on the prediction of phenotypic traits in potato using multi-omics data [67], integration of data layers did not enhance Random Forest model accuracies much more than when using individual layers. Layer contributions to trait prediction accuracies will undoubtedly depend on the biological system under investigation. In the current study, although many inter-layer covariate correlations exist, the substantial gain made by integrating the datasets indicate that some of these layers harbour unique information and are not redundant towards prediction of growth performance traits in fish. To summarise, considerable improvements in trait prediction accuracies were made by utilising all data rather than any single data modality alone. 

### 3.5. Important Random Block Predictor Variables for Feed Efficiency

Here we focus attention on the FE-specific models that were constructed in Figure 4, and interrogate them to identify a subset of the most predictive features. In Random Forest (RF) regression, the measure of ‘variable importance’ is used for feature selection. Analogous to the distinction between predictive importance and explanatory/causal importance in conventional regression models, RF-based feature selection serves two objectives: (1) to identify a smaller subset of variables sufficient for response/trait prediction, and (2) to identify important variables highly related to the response/trait for explanatory and interpretation purposes. For the first objective, RF is highly applicable for constructing parsimonious trait prediction models since the algorithm is resilient to overfitting, is not impacted by high predictor multicollinearity, has versatility towards different variable types, is relatively robust to outliers, and can efficiently handle missing data and non-linear parameters. To enable biologically relevant interpretations for the second objective, RF has some limitations since magnification of all explanatory variables, including those with high redundancy, would ideally be achieved. Collinear variables which may be associated with a trait of interest can become down weighted in their variable importance scores if they do not enhance predictive accuracy, which means the full extent of informative features are often not realised [68]. Although variables which were most important to the accuracies of the Random Block models in predicting FE may only be a subset of those with significant biological meaning, functional evaluation of their roles are however warranted.

**Figure 7:**
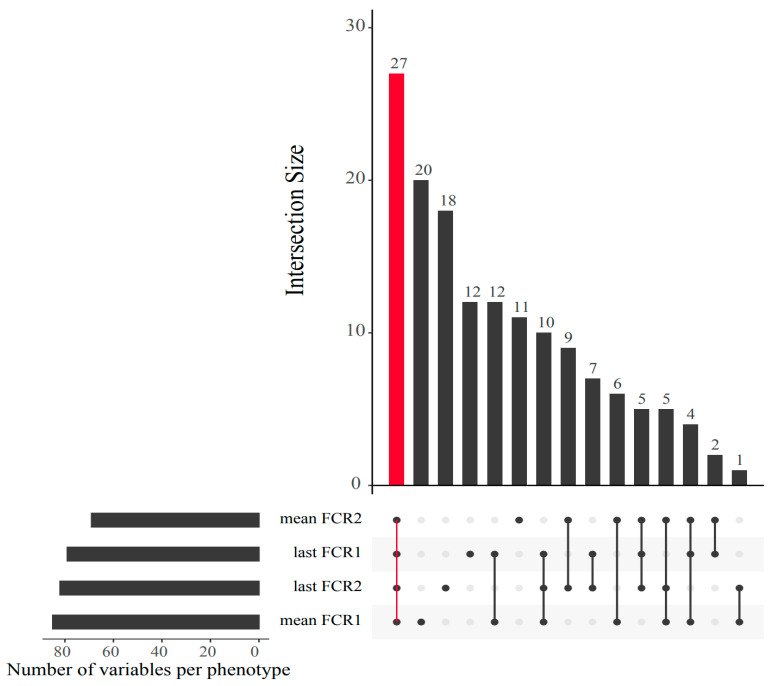
UpSet plot displaying shared predictor covariates between the best Random Block model for each of the four feed efficiency measures. The bar highlighted in red shows the number of covariates shared across all feed efficiency measures.

**Figure 8:**
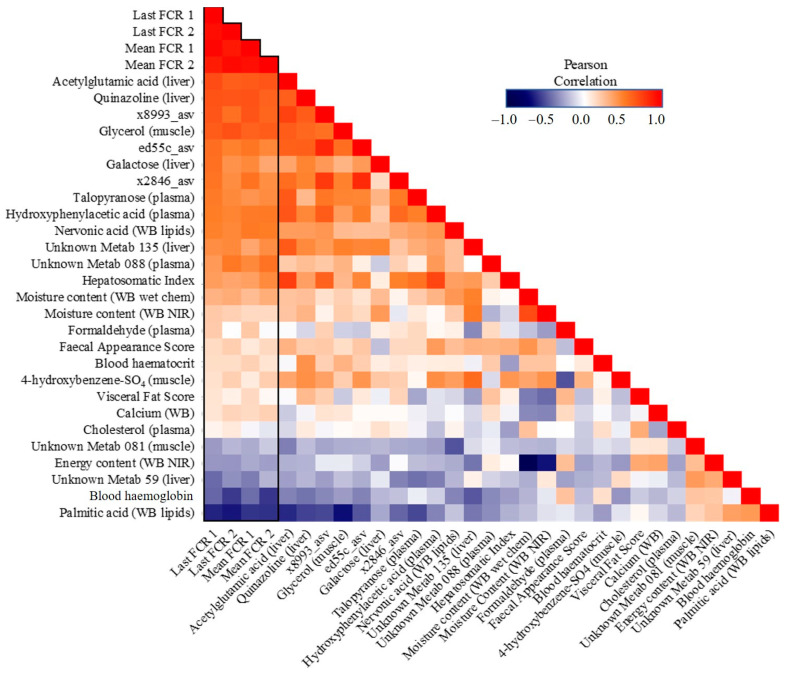
Correlation heatmap of important shared features (N = 27) in the Random Block prediction models for feed efficiency across all four measures.

**Figure 9:**
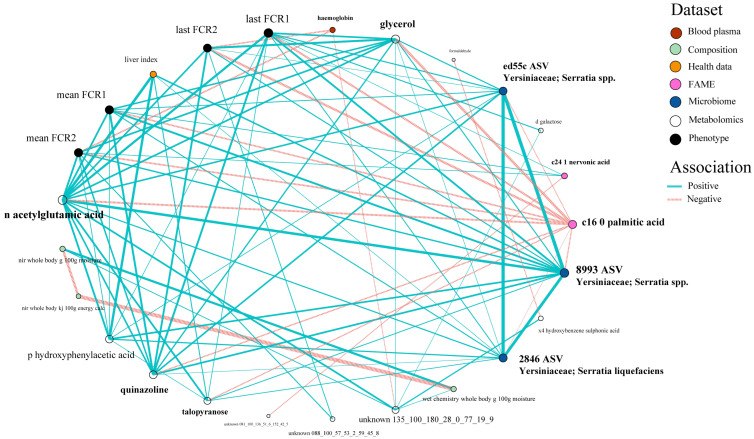
Associations between the 27 covariates which were consistently selected by the Random Block models in predicting FE, visualised via a trimmed co-occurrence network highlighting 21 major relationships.

Looking more closely at the highest ranked co-occurring variables important to the best-performing Random Block models for FE, scope exists to gain mechanistic insight into FE variation. Many of these important predictor variables (N = 27) which originated from the integrated data were shared among all four FE trait prediction models (Figure 7). Interactions between this subset of variables and FE values were further assessed via correlation analyses (Figure 8) and a co-occurrence network (Figure 9), which additionally highlight some feature associations across the different data layers. These analyses notably show that three bacterial ASVs (i.e., ‘x8993’, ‘ed55c’ and ‘x2846’) from the family *Yersiniaceae* (genus *Serratia*) were negatively correlated with overall mean FE (*r* = −0.51 to −0.64; *p*-values < 0.01). *Serratia* spp. also formed positive correlations with N-acetylglutamic acid, hepatosomatic/liver index, and quinazoline, and a negative correlation with palmitic acid; all are significantly correlated to FE values. Members of *Yersiniaceae* are known fish pathogens [69,70,71,72,73], and their higher abundance in faeces of salmon with poor FE may indicate the existence of an intestinal infection/disease-related association.

Putative links between some clinical covariates and FE in fish were signalled, although weak–moderate linear correlations with the trait may be suggestive of more complex relationships. FE predictors selected as important by the Random Block models included hepatosomatic index, visceral fat score, faecal appearance score, plasma cholesterol, blood haemoglobin, and haematocrit value. Interestingly, haemoglobin and haematocrit were recently identified as potential indicators of FE phenotypes in swine, with mechanistic underpinnings involving oxygen carrying capacity [74]. Further analysis of these parameters is warranted to substantiate links with FE in fish. All clinical covariates measured in this study were considered within the typical range.

Muscle glycerol showed a negative association (*r* = −0.66; *p* < 0.001) with overall mean FE. Higher glycerol concentrations in fish with poor FE may suggest increased energy utilisation of lipolysis substrates. Glycerol is currently receiving much attention as a feed supplement for the aquaculture of various fish species, with a proposed role in substrate provision for gluconeogenesis; thus, outcompeting dietary amino acids so they are more available for other physiological functions, such as growth [75,76]. Lower glycerol levels in fish with better FE may also reflect its increased substrate use for energy production, and differential turnover and partitioning of this metabolite should be considered an area for further study.

Levels of liver N-acetylglutamic acid (NAG) were also negatively associated (*r* = −0.68; *p* < 0.001) with FE. NAG is an essential allosteric cofactor for the first enzyme of the urea cycle in higher vertebrates. Most teleost fish directly clear the majority of nitrogenous waste as ammonia though the gills; however, *Oncorhynchus* spp. are known to produce ornithine–urea cycle enzymes as a supplementary route for nitrogen excretion and arginine synthesis [77,78,79]. FE in poultry can be modulated by NAG supplementation [80] and dietary N-carbamylglutamate, a metabolically stable analogue of NAG, improves protein efficiency ratios and growth in fish [81,82]. Variation in liver NAG may reflect a link between nitrogenous waste removal processes and FE in *O. tshawytscha*. This finding led us to look for a potential waste product relationship; levels of liver urea were also negatively correlated (*r* = −0.68; *p* < 0.001) with FE which supports a nitrogenous-based link. Urea excretion has a higher energetic cost than clearance of ammonia which may partly underpin this inverse association with FE.

Urea production in some fish taxa can occur through three routes: the ornithine-urea cycle (ammonia detoxification), the uricolytic pathway (purine degradation), and the argininolytic pathway (degradation of dietary arginine). Relative contributions of these processes towards ureagenesis depend on species [83]. We screened the liver proteomics data layer for proteins relevant to urea production, but no significant associations were identified between enzyme abundances and FE. Mechanistic foundations for the urea variations we observed in the current study should focus on enzyme activities.

Quinazoline is a predominant scaffold in many naturally occurring bioactive alkaloids, and levels in the liver of *O. tshawytscha* were negatively associated with overall mean FE (*r* = −0.68; *p* < 0.001). Marine sources of quinazoline derivatives are diverse, being synthesised by numerous bacterial, fungal, and marine invertebrate taxa, and a few fish species [84]. However, a functional role for this metabolite in salmon FE is currently uncertain.

Key fatty acid (FA) constituents of whole-body lipids which were associated with FE were nervonic acid (C24:1[n-9]) and palmitic acid (C16:0). Nervonic acid, a sphingomyelin-enriched long chain monounsaturated FA common in fish oils [85,86], displayed a negative relationship with FE (*r* = −0.51; *p* < 0.01). Nobrega et al. [87] recently identified a negative association between nervonic acid content and daily feed intake in Nile tilapia and suggested that selective incorporation of this FA into cell membranes during periods of food limitation may aid in winter survival, although a mechanism was not proposed. Whole-body palmitic acid content was positively associated (*r* = 0.69; *p* < 0.001) with FE. Palmitic acid is abundant in the diet of farmed salmon and is constituently used as a saturated pairing to long-chain polyunsaturated FAs in polar lipids.

## 4. Conclusions

Improving feed efficiency (FE) is a strategy to reduce production costs and improve sustainability in fish aquaculture. Measurement of individual FE in fish is logistically challenging, time-consuming, and costly. The identification of reliable predictors of FE could reduce phenotyping efforts. FE is governed by a myriad of intrinsic and environmental factors, which multi-omic analyses are anticipated to contribute greater mechanistic understanding. This study demonstrated the novel use of the Random Block algorithm to integrate a diverse set of multimodal covariates in a salmonid (*Oncorhynchus tshawytscha*) model to predict a range of growth performance metrics, including FE. Our findings revealed the existence of strong concordance among covariate layers and demonstrated the unique contributions each layer provides towards trait prediction accuracies. FE-associations were made with some bacterial taxa, metabolites, and biochemical pathways, pointing towards key areas for subsequent hypothesis testing. Results further emphasise that FE is a highly complex trait; no single parameter we measured was able to reliably predict FE alone, but rather necessitated multiple variables for accurate prediction. Random Forest-based algorithms are useful for integrating multiple omics and clinical covariates and provide unique application for predicting growth and efficiency phenotypes in farmed Chinook salmon.

## Figures and Tables

**Table 1:** Summary of explanatory variables used in this study for the prediction of feed efficiency and performance-based traits in fish (ASVs = amplicon sequence variants; F = faeces; L = liver; M = white muscle; P = plasma; WB = whole body homogenate; Fi = fillet; B = blood).

Covariate Class		Feature Type	Sample Type	*N* Layers	*N* Features
Non-clinical	Microbiome	Microbial ASVs	F	1	135
	Proteome	Proteins	L, M	2	3223
	Metabolome	Metabolites	L, M P	6	556
	General composition	Macromolecules	WB, Fi	1	28
	Lipid composition	Fatty acids	WB	1	32
Clinical	Blood biomarkers	Proteins and Metabolites	B	1	24
	Haematology indices	Blood cell profiles	B	1	12
	Health indices	Organs	O	1	5
Total				14	4077

## Data Availability

The data that support the findings of this study are available on reasonable request from the corresponding authors. The data are not publicly available due to privacy.

## Supplementary Materials

The following supporting information can be downloaded at: https://www.mdpi.com/article/10.3390/biology12081135/s1, Methods S1: Experimental design, Methods S2: Microbiomics, Methods S3: Proteomics, Methods S4: Metabolomics, Methods S5: General composition, Methods S6: Fatty acids, Methods S7: Blood biomarkers, and Methods S8 Haematology.
