# Supplementary material for: Prediction of Feed Efficiency and Performance-Based Traits in Fish via Integration of Multiple Omics and Clinical Covariates
Source: Biology (Basel). 2023 Aug 15;12(8):1135. doi: 10.3390/biology12081135 (PMC10452023; doi:10.3390/biology12081135)
Supplement: Supplementary file 1 [file biology-12-01135-s001.zip › biology-2430619-supplementary/Methods S1 - Experimental Design.pdf]

Prediction of feed efficiency and growth traits in fish via integration of multiple omics and clinical covariates

Young et al. 2023

Supplementary Methods 1

1. Experimental design

1.1 Fish and Recirculating Aquaculture System

Approximately 1620 juvenile (ten-month-old) Chinook salmon (sourced from Clearwater Hatchery [Mount Cook Alpine Salmon Ltd]) were individually tagged with a unique passive integrated transponder (PIT) tags (HIDGlobal, EM4305 684,230, 12 mm glass tags). The PIT tag number was used in all data collections to identify the fish and was used to link data collections together. Fish were acclimated to tank conditions at 15°C (for a period of 21 to 24 days), transported to the Cawthton Aquaculture Park Finfish Research Centre (CAP-FRC; Nelson, New Zealand), and evenly distributed across nine 8000L tanks on a freshwater Recirculating Aquaculture System (RAS). Each tank was set up with two oxygen diffusers, a dissolved oxygen probe, water inlet, outlet in the middle of the tank bottom, and covered with a black net. The maximum density was below 25 $kg.m^3$ throughout the experimental period. Population parameters (i.e., forklength, body weight, condition factor) were tested for homogeneity (Chi-square tests; p-values > 0.05) among the tanks.

The RAS was supplied with fresh water from the local town water supply that had been carbon filtered to remove any residual chlorine. Each tank was set up with an oxygen diffuser, a dissolved oxygen probe, water inlet, outlet in the middle of the tank bottom and covered with a black net. The oxygen was maintained at above 6.1 mgL^{-1} (above the saturation of 63%). The outflow water from each tank was treated in six steps: 36 μm drum filter (Faivre 4/80 series), moving bed biofilm reactor (16 m^3 per system), foam fractionator (RK2 RK150) injected with ozone, oxygen injection, UV disinfection (Ultra Aqua UV: minimum dose of 60 $mg.cm^2$) and temperature adjustment to ensure sufficient waste removal, reoxygenation and disinfection of the wastewater. 5–10% of the water in the system was replaced per day by newly filtered (100 μm) and UV-treated freshwater. A 24 hour lighting regime was applied. The water treatment system was designed and supplied by Fresh by Design Ltd (Moss Vale, NSW, Australia).

1.2 Diet

Differing feed rations were implemented to partly influence feed efficiency (FE) and thus provide fish with varying FE at the end of the trial for sampling. Triplicate tanks were assigned to one of three feeding conditions (dietary rations at 100%, 80%, and 60% of satiation). Treatment rations

were calculated based on the average number/mass of food pellets consumed by the 100% ration group, with the restricted rations being adjusted daily based on the previous day's feed intake. As growth was different among the three ration groups, the 60% and 80% rations were determined based on the percentage of the amount fed to the 100% ration tanks when they were of similar weight.

The fish were fed 4 mm and 6 mm Tasman Freshwater diet (22.2% fat, 43.4% protein, 20.7% carbohydrate, 5.3% moisture and 8.4% ash) manufactured specifically for the trial by Ridley using their new experimental extruder. The 4 mm pellets were fed from the start of the trial until 30 Jan 2019. A mix of 4 mm and 6 mm pellets, with increasing proportions of the 6 mm pellets, was fed over a four-day transition period, and from then to the end of the trial only 6 mm pellets were used. One meal per day was fed to all fish at the same time each day. Fish were reared under this feeding regime for approximately eight months.

1.3 Sampling and Assessments

During the experiment, fish were measured on four occasions (Days 35, 80, 130 and 190). At each assessment, all fish were weighed, fork length measured to the nearest 0.1 cm, and individual feed intake measured by X-ray. Fish were anesthetized using 65 ppm tricaine methanesulfonate (Syndel, Canada) with adequate oxygen levels during these assessments, and PIT tags were scanned into the computer using a microchip tag reader (Avid-Power TracKer VI, Noosaville Bc, Queensland, Australia).

A X-radiography method was adapted to measure individual daily feed intake (DFI) on each assessment. Feed containing 0.5 mm X-ray opaque "ballotini" beads (ceramic zirconium silicate type ZS, 9305, 0.4–0.6 mm SiLibeads®, Sigmund Lindner GmbH, Warmensteinach, Germany), at a 0.5% inclusion rate were used to quantify feed intake. For each feed, a series of samples, of known weight, were X-rayed. The number of beads in each sample was counted using semi-automated bead counting software "Bead Counter" developed by AgResearch, validated using manual counts, and plotted against the weight of the sample (intercept at 0) to provide a diet-specific calibration curve (R^2 value was higher than 0.97% for both diets). The X-ray images were obtained using an Atomscope HFX90V EX9025V portable X-ray Unit (DLC Australia Pty Ltd., Melbourne, Australia) and Canon CXDI 410C Wireless Cesium Amorphous Silicon digital radiographic receptor (DLC Australia Pty Ltd., Melbourne, Australia). Whole anesthetized fish were then X-rayed to determine the total number of ballotini in the gastrointestinal tract.

Actual feed consumption was determined each day and the number of uneaten pellets was recorded for each tank 15 min after feeding using a swirl separator and a sieve of smaller mesh size (3 mm) than the pellet size (initial pellet size was 4 mm). After dehydration and removing dust particles, the pellets were counted using a pellet counter (Contardor2, PFEUFFER GMBH, Kitzingen, Germany).
