# Supplementary material for: Prediction of Feed Efficiency and Performance-Based Traits in Fish via Integration of Multiple Omics and Clinical Covariates
Source: Biology (Basel). 2023 Aug 15;12(8):1135. doi: 10.3390/biology12081135 (PMC10452023; doi:10.3390/biology12081135)
Supplement: Supplementary file 1 [file biology-12-01135-s001.zip › biology-2430619-supplementary/Methods S2 - Microbiomics.pdf]

Prediction of feed efficiency and growth traits in fish via integration of multiple omics and clinical covariates

Young et al. 2023

Supplementary Methods 2

1. Microbiomics

1.1 Sample Preparation

Bacterial DNA was extracted directly from ~200mg faecal samples using the NucleoSpin Soil kit (Macherey-Nagel; Düren, Germany) and following the manufacturer's instructions. DNA concentrations were measured using a NanoPhotometer® NP80 spectrophotometer (Implen; Munich, Germany). Extracted DNA was maintained in the elution buffer and stored at −80°C for until analysis.

1.2 PCR amplification for 16S RNA gene amplicon sequencing

The bacterial universal primers 27F (V1, 5′ AGA GTT TGA TCM TGG CTC AG 3′) and 519R (V3, 5′ GWA TTA CCG CGG CKG CTG 3′), including a 12-base Golay barcode as described by Caporaso et al. (2012), were used for conducting Polymerase Chain Reaction (PCR). To evaluate the bacterial DNA, 2 × 300 bp pair-ended amplicon sequencing of the V1–V3 region of the 16S rRNA gene was performed using a MiSeq platform (Illumina; San Diego, California, USA) at the Ramaciotti Centre for Genomics (RCG; Kensington, NSW, Australia). Sequencing data were trimmed by removing the primer, bar code and adapter regions using internally developed algorithms by RCG. Paired-end sequences were joined using FASTQ-join (Aronesty 2011) and generated as demultiplexed FASTQ files for further analysis. Sequences were sorted by individual sample and filtered by removing the low-quality reads.

1.1 Bacterial identifications

Taxonomic analyses of sequence reads were further processed by the latest version of the Seed 2 pipeline (Seed v.2.1) (Větrovský et al. 2018). Sequence alignment, denoising, chimera check and clustering were carried out by using Seed 2 external programs comprising USEARCH v7.0.1090, MAFFT v7.215, and MOTHUR v1.34.4. The sequences for each cluster were then sorted by length and clustered with a 3% divergence cut-off to define operational taxonomic units (OTU) from centroids. Clusters with fewer than two reads and reads <100 bp were excluded, followed by further clustering at a 3% divergence level using USEARCH to optimise the final consensus sequences accurately and define OTUs. Classification of OTUs was performed against the SILVA non-redundant 16S rRNA database (SILVA SSU 138 [Quast et al. 2013]; accessed Dec 16th 2019).

2. References

Caporaso, J., Lauber, C., Walters, W., Berg-Lysons, D., Huntley, J., Fierer, N., Owens, S.M., Betley, J., Fraser, L., Bauer, M., Gormley, N., Gilbert, J.A., Smith, G., Knight. R. 2012. Ultra-high-throughput microbial community analysis on the Illumina HiSeq and MiSeq platforms. *ISME Journal*, 6, 1621–1624.

Aronesty, E. 2011. *Command-Line Tools for Processing Biological Sequencing Data*. Expression Analysis, A Quintiles Company, Durham, NC, USA

Vetrovský T, Baldrian P, Morais D. 2018. SEED 2: A user-friendly platform for amplicon high-throughput sequencing data analyses. *Bioinformatics*, 34(13), 2292–2294.

Quast C, Pruesse E, Yilmaz P, Gerken J, Schweer T, Yarza P, Peplies J, Glöckner FO. 2013. The SILVA ribosomal RNA gene database project: improved data processing and web-based tools. *Nucleic Acids Research*, D590–6.
