# Supplementary material for: Prediction of Feed Efficiency and Performance-Based Traits in Fish via Integration of Multiple Omics and Clinical Covariates
Source: Biology (Basel). 2023 Aug 15;12(8):1135. doi: 10.3390/biology12081135 (PMC10452023; doi:10.3390/biology12081135)
Supplement: Supplementary file 1 [file biology-12-01135-s001.zip › biology-2430619-supplementary/Methods S3 - Proteomics.pdf]

Prediction of feed efficiency and growth traits in fish via integration of multiple omics and clinical covariates

Young et al. 2023

Supplementary Methods 3

1. Proteomics

1.1 Sample preparation

Livers and muscles (~13 mg of wet tissue) from each fish were individually homogenised for 10–15 sec in Eppendorf tubes containing denaturation buffer (7 M urea, 2 M thiourea, in 40 mM Tris; pH 8) and EDTA-free protease inhibitor cocktail (Roche; Basel, Switzerland) using Tissue Tearor Homogenizer (Biospec Products; Oklahoma, USA). The protein sample was diluted to approximately 1 $mg.mL^{-1}$ in denaturation buffer (7 M urea and 2 M thiourea in 40 mM Tris; pH 8.0) according to the results of the Pierce A660 spectrophotometric assay (Thermo Scientific; Rockford, Illinois, USA). Aliquots of 30 µg protein were sequentially reduced using 10 mM Dithiothreitol (Cytiva; Massachusetts, USA) overnight at 4°C, alkylated using 50 mM iodoacetamide (Cytiva; Massachusetts, USA) for 2 hrs at ambient temperature and then digested with 1.2 µg proteomics-grade trypsin/LysC (Promega; Wisconsin, USA) according to the SP3 protocol (Hughes et al., 2019). Digests were acidified by the addition of trifluoroacetic acid to 0.1% and peptides collected by centrifugation at 21,000 rcf for 20 min. Samples were further cleaned up by offline desalting using ZipTips (Merck Millipore; Cork, Ireland) according to manufacturer's instructions.

1.2 High-pH reversed phase HPLC peptide fractionantion

Two experiment-specific peptide spectral libraries for liver and muscle protein extracts were generated using off-line high-pH fractionation. For each library, a pooled digest comprising aliquots of each sample set (180 µg total) was desalted using Pierce desalting spin columns (Thermo Scientific, Rockford IL, USA) according to manufacturer's guidelines. Each sample was evaporated to dryness, then resuspended in 25 µL HPLC loading buffer (2% acetontrile containing 0.05% TFA) and injected onto a 100 × 1 mm Hypersil GOLD (particle size 1.9 µm) HPLC column (Thermo Scientific; Massachusetts, USA). Peptides were separated using an Ultimate 3000 RSLCnano system (Thermo Scientific; Massachusetts, USA) with micro-fractionation, and automated sample concatenation enabled, operated at 30 $mL.min^{-1}$ using a 40 min linear gradient of 96% mobile phase A (water containing 1% triethylamine, adjusted to pH 9.6 using acetic acid) to 50% mobile phase B (80% acetonitrile containing 1% triethylamine), followed by 6 min washing in 90% B and re-equilibration in 96% A for 8 min. Sixteen concatenated fractions were collected into 0.5 mL Protein lo-bind Eppendorf tubes, evaporated to dryness then reconstituted in HPLC loading buffer.

1.3 Mass spectrometry – data-dependent acquisition (DDA)

Peptide fractions were analysed by nanoflow HPLC-MS/MS using an Ultimate 3000 RSLCnano system (Thermo Scientific; Massachusetts, USA) coupled with a Q-Exactive HF mass spectrometer fitted with a nanospray Flex ion source (Thermo Fisher Scientific; Massachusetts, USA) and controlled using Xcalibur software v4.3 (Thermo Scientific; Massachusetts, USA). Approximately 1 mg of each fraction was injected and separated using a 90 min segmented gradient by pre-concentration onto a 20 mm × 75 mm PepMap 100 C18 trapping column, then separation on a 250 mm × 75 mm PepMap 100 C18 analytical column at a flow rate of 300 nL.min^{-1} and held at 45°C. MS Tune software v2.9 (Thermo Scientific; Massachusetts, USA) parameters used for data acquisition were: 2.0 kV spray voltage, S-lens RF level of 60, and heated capillary set to 250°C. MS1 spectra (390–1500 m/z) were acquired at a scan resolution of 120,000 followed by MS2 scans on precursors selected according to a Top15 DDA method, with 30 sec dynamic exclusion of fragmented peptides. MS2 spectra were acquired at a resolution of 15,000 using an AGC target of 2×10^5, maximum IT of 28 ms, and normalised collision energy of 30.

1.4 Mass spectrometry - data-independent acquisition (DIA)

Individual peptide samples were analysed by nanoflow HPLC-MS/MS using the instrumentation and LC gradient conditions described above but using DIA mode. MS1 spectra (390–1240 m/z) were acquired at 120k resolution, followed by 26 × 25 amu sequential DIA MS2 scans over the range of 397.5–1027.5 m/z, with 1 amu overlap between windows. MS2 spectra were acquired at a resolution of 30,000 using an AGC target of 1×10^6, maximum IT of 55 ms, and normalised collision energy of 27.

1.5 Raw data processing

Both DDA- and DIA-MS raw files were processed using Spectronaut software, version 13.12 (Biognosys AB; Schlieren, Switzerland). Each sample-specific library was generated using the Pulsar search engine to search DDA MS2 spectra against the NCBI *Oncorhynchus tshawytschai* protein sequence database (txid 74940; comprising 74,180 entries at 26/08/2019). Spectral libraries were generated using BGS factory settings in Spectronaut software, including N-terminal acetylation and methionine oxidation as variable modifications and cysteine carbamidomethylation as a fixed modification, up to two missed cleavages allowed and peptide, protein and PSM thresholds set to 0.01. Retention time alignment was based on the high-precision iRT concept (Bruderer et al. 2016). With the exception that single-hit proteins were excluded, BGS factory settings were used for protein identification and relative quantitation between samples, using global normalisation based on median MS2 intensity values.

1.6 Post-processing data analysis

Spectronaut protein quantitation pivot reports, including protein description, gene names, and NCBI accession numbers, from all individuals (26 fish) were uploaded into Perseus software v1.6.10.50 (http://www.coxdocs.org/doku.php?id=:perseus:start) for further data processing and statistical analysis. Quantitative values were $\log_2$ transformed, and proteins filtered according to the number of valid values. Proteins detected in <70% of samples were excluded from further analysis,

and remaining missing values were imputed with random intensity values for low-abundance proteins based on a normal abundance distribution. Batch effects were removed via the ComBat method (Adjusting batch effects in microarray expression data using empirical Bayes methods) using MetaboAnalyst v4.0 software (Chong et al. 2018). Chinook salmon proteins were annotated against Zebrafish (*Danio rerio*) using BlastP (https://blast.ncbi.nlm.nih.gov/Blast.cgi?PAGE=Proteins).

2. References

Bruderer, R., Bernhardt, O.M., Gandhi, T. and Reiter, R., 2016. High-precision iRT prediction in the targeted analysis of data-independent acquisition and its impact on identification and quantitation. *Proteomics*, 16: 2246–2256.

Chong, J., Soufan, O., Li, C., Caraus, I., Li, S., Bourque, G., Wishart, D.S. and Xia, J., 2018. MetaboAnalyst 4.0: Towards more transparent and integrative metabolomics analysis. Nucleic Acids Research, 46(W1), W486–W494.

Hughes, C.S., Moggridge, S., Müller, T., Sorensen, P.H., Morin, G.B. and Krijgsveld, J., 2019. Single-pot, solid-phase-enhanced sample preparation for proteomics experiments. *Nature Protocols*, 14: 68–85.
