# Supplementary material for: Prediction of Feed Efficiency and Performance-Based Traits in Fish via Integration of Multiple Omics and Clinical Covariates
Source: Biology (Basel). 2023 Aug 15;12(8):1135. doi: 10.3390/biology12081135 (PMC10452023; doi:10.3390/biology12081135)
Supplement: Supplementary file 1 [file biology-12-01135-s001.zip › biology-2430619-supplementary/Methods S4 - Metabolomics.pdf]

Prediction of feed efficiency and growth traits in fish via integration of multiple omics and clinical covariates

Young et al. 2023

Supplementary Methods 4

1. Metabolomics

1.1 Metabolite extraction

Frozen plasma (100 µL) was slowly thawed on ice and mixed with two internal standards: 20 µL of 10 mM L-alanine-2,3,3,3-*d*4 relevant for methyl chloroformate (MCF) derivatisations, and 20 µL of 10 mM Ribitol for relevant for trimethyl silylation (TMS) derivatisations. Prior to extraction, plasma samples were dried for 4 h at 0°C under vacuum (Thermo Scientific™ Express SC250EXP SpeedVac™ Concentrator System with a Refrigerated Vapor Trap). Metabolites and internal standard were co-extracted using a two-step sequential extraction and a methanol-water solvent system (MeOH [Merck; Darmstadt, Germany]; Milli-Q filtered H_2O): 1000 µL of cold (–20°C) 50% MeOH solution was added to the dried samples, vortexed for 1 min, then centrifuged for 5 min at 20,800 rcf and –9°C. The process was repeated with 1000 µL cold 80% MeOH solution and the supernatants combined. For muscle and liver tissues, samples were first lyophilised (Christ Alpha 2–4 LD plus) for 24 hrs to constant mass, ground with a mortar and stainless-steel micro-pestle, accurately weighed to 10 mg (±2mg), and combined with 20 µL of each internal standard; extractions were then performed in a similar manner as with the dried plasma samples. For quality assurance (QA) purposes, a homogenate was prepared for each sample matrix type by mixing equal volumes (50 µL) of all extracts; sub-aliquots of these homogenates provided replicate sets of pooled quality control (*p*QC) samples. All sample extracts were dried (SpeedVac™ Concentrator) and stored at –80°C until derivatisation.

1.2 Metabolite derivatisation

MCF derivatives were prepared using a modified version of the alkylation procedure described in Smart et al. (2010) to convert amino and non-amino organic acids into volatile carbamates and esters, thus obtaining a broad spectrum of stable analytes (Villas-Bôas et al. 2011). Dried extracts were re-suspended in 400 µL of 1 M sodium hydroxide (Merck) and 68 µL of pyridine (Sigma-Aldrich). Mixtures were transferred to Kimble™ silanized borosilicate glass tubes (12 × 75 mm) (Thermo Fisher; Auckland, NZ) containing 334 µL of methanol. 40 µL of MCF reagent (Sigma-Aldrich) were added and samples were vortexed for 30 sec. Another 40 µL of MCF was added, followed by vortexing for 30 sec. To separate the MCF derivatives from the mixture, 400 µL of chloroform (Merck) were added, vortexed for 10 sec, then followed by addition of 800 µL of 50 mM sodium bicarbonate (Merck) solution and vortexed for a further 10 sec. The mixture was centrifuged (5810R; Eppendorf AG; Hamburg, Germany) for 5 min at 1174g and 6°C. The upper aqueous layer

was discarded and a small amount of anhydrous sodium sulphate (BDH Chemicals; Poole, UK) was added to remove residual H_2O. The chloroform phase containing the MCF derivatives was transferred to 2 mL amber CG glass vials fitted with inserts (Sigma-Aldrich). A separate standard amino acid mix (100 µl, 20 mM [Merk]) and a sample blank containing 20 µL of L-alanine-2,3,3,3-*d*4 were similarly derivatised for QC purposes.

TMS derivatives were prepared following Pinu et al. (2014). Freeze-dried extracts were resuspended in 80 µL of methoxyamine hydrochloride solution in pyridine (2 mg/100 mL), vortexed for 1 min, transferred to 2 mL GC vials, and incubated at 30°C for 90 min. 80 µL of N-methyl-N (trimethylsilyl)trifluoroamide was added to the mixture, vortexed for 1 min, and further incubated at 37°C for 30 min to complete the derivatization reaction. Sample blanks and *p*QC's were similarly prepared.

1.3 Gas chromatography mass spectrometry analysis

Derivatised samples were injected into a GC-MS system (Thermo Trace GC Ultra system coupled to an ISQ mass selective detector [EI] operated at 70 eV [Thermo Fisher Scientific Inc.; New York, USA]), which was fitted with a ZB-1701 GC capillary column (30 m × 250 µm id × 0.15 µm with 5 m stationary phase [86% dimethylpolysiloxane, 14% cyanopropylphenyl]) (Phenomenex; Torrance, California, USA). The analysis parameters were conducted according to Smart et al. (2010). Samples were injected using a CTC PAL autosampler into a Siltek™ 2 mm id straight unpacked inlet liner under pulsed splitless mode with the injector temperature at 260°C. The helium gas flow through the GC-column was set at a constant flow of 1 $mL.min^{-1}$. The GC-oven temperature was initially held at 45°C for 2 min, and then raised with a gradient of 9°C min^{-1} to 180°C; after 5 min the temperature was increased at 40°C min^{-1} to 220°C. After a further 5 min, the temperature was increased at 40°C min^{-1} to 240°C and held for 11.5 min. Finally, the temperature was increased at 40°C min^{-1} until it reached 280°C where it was held for a further 2 min. The interface temperature was set to 250°C and the quadrupole temperature was set at 230°C. The mass spectrometer was operated in scan mode, starting after 5.5 min, with mass range of 38–550 amu and a scan time of 0.1 sec.

As part of the general QA strategy, various QC samples were incorporated into the analytical workflows. Injections of the following were made in sequence at the beginning of each days' GC-MS run: pure chloroform, a non-derivatised standard alkane mix, a derivatised metabolite standard mix, and a sample blank – thus enabling system checks for column carry over, instrument stability, overall method reliability, and contamination stemming from solvents/reagents/equipment, respectively. The 37 fish used for this integrated 'omics study came from a wider project involving a larger set of metabolomics samples (N = 108 for each of the three sample-type matrices). Thus, monitoring and adjusting for technical variation associated with batch effects was important. As such, MCF samples were prepared and injected in seven sub-batches over seven consecutive days. Samples were completely randomised and *p*QC's were injected at the beginning (after the sample blank and before the first real sample), middle (after eight real samples), and end (after another eight real samples) of each day. This strategy resulted in a total of 21 *p*QC injections for the MCF runs. TMS samples were also prepared in a randomised sequence, but injected over 14 smaller half-day sub-batches due to the lower stability of the TMS-derivatised products. *p*QC's were injected at the beginning and end of each TMS sub-batch (with eight real samples in between). This strategy resulted in a total of 28 *p*QC injections for the TMS runs.

1.4 Spectral processing and metabolite identifications

Metabolite data extraction and analysis were undertaken based on protocols described in Smart et al. (2010). Deconvolution of chromatographic data was performed using the Automated Mass Spectral Deconvolution and Identification System (AMDIS v2.66), online software distributed by the National Institute of Standards and Technology (NIST) (http://www.amdis.net/). Metabolite identifications and peak integrations (relative quantification) were conducted using Chemstation Software (Agilent Technologies) and customised R xcms-based scripts (Aggio et al. 2011) to interrogate in-house libraries of MCF- and TMS-derivatised compounds constructed using pure standards. Compound identifications were based on matches to both the MS spectrum of the derivatised metabolite and its respective chromatographic retention time. The NIST mass spectral library was used for additional assignments. Semi-quantification data were generated from the maximum height of the reference ion for the compound peak. The reference ion used as a measure of abundance for each compound is usually the most abundant fragment and is not the molecular ion. MassOmics v2.3 (https://rdrr.io/github/MASHUOA/MassOmics/), a windows-based data extraction application, was used to generate a composite list of all metabolites detected in the dataset. This report contained metabolite identifications, mass spectral identification scores, the most abundant ion for each library match, the number of times each metabolite was detected in the whole dataset, and the amount of retention time drift for each metabolite. Analyses were carried out in the 'R' platform v3.3.1 (http://www.r-project.org/).

1.5 Data pre-processing

A Microsoft® Excel file containing peak height data for each metabolite was generated and manually screened to filter repeats (keeping entries with highest match factors). Technical variations introduced during sample preparation and GC-MS analysis were accounted for using Systematic Error Removal using Random Forest (SERRF), a *p*QC-based normalisation procedure designed for multi-batch metabolomics data (Fan et al. 2020). For each compound, SERRF uses the *p*QC data to build an RF model for estimating systematic error (e.g. batch effects, day-to-day variation). The models are then applied to the study samples to account for this error. The benefits of SERRF over traditional *p*QC-based normalisation approaches is that the systematic error is assumed to be associated with the behaviour of other compounds in addition to batch effects and injection order influences. Data were subsequently subjected to an 8-step QA regime: (1) normalisation to the peak height of the relevant internal standard (i.e., *d*4-alanine for MCF derivatives, ribitol for TMS derivatives) to compensate for other variation sources during extraction (e.g. variable metabolite recoveries), (2) blank-correction (i.e., subtraction of the average peak responses in sample blanks from the datasets) to remove background noise and/or signals from contaminants, (3) deletion of non-positive data (i.e., values ≤0 after blank-correction), (4) elimination of obvious sources of contamination (i.e., derivative artefacts and compounds with non-biological origins via manual examination), (5) normalisation to sample-specific biomass (for tissue data), (6) condensing feature parallels (e.g., secondary peaks, discrete sub-type derivatives of parent compounds) as appropriate by merging peak abundances (i.e., sum of peaks when peak $X_{2\ldots n} \geq {}^{X_1}/_5$) or through data elimination (i.e., when peak $X_{2\ldots n} \leq {}^{X_1}/_5$), (7) integration of feature overlaps between MCF and TMS datasets in matched samples by removing commonly-derivatised amino and non-amino organic acid data (n=32)

from the TMS dataset for reliability purposes (see Villas-Bôas et al. 2011), and finally, (8) replacing missing values using MetaboAnalyst v4.0 software (Chong et al. 2018) via the 'half minimum' estimation approach.

2. References

Aggio, R., Villas− Bôas, S.G. and Ruggiero, K., 2011. Metab: An R package for high-throughput analysis of metabolomics data generated by GC-MS. *Bioinformatics*, 27(16), 2316–2318.

Chong, J., Soufan, O., Li, C., Caraus, I., Li, S., Bourque, G., Wishart, D.S. and Xia, J., 2018. MetaboAnalyst 4.0: Towards more transparent and integrative metabolomics analysis. Nucleic Acids Research, 46(W1), W486–W494.

Fan, S., Kind, T., Cajka, T., Hazen, S.L., Tang, W.W., Kaddurah-Daouk, R., Irvin, M.R., Arnett, D.K., Barupal, D.K. and Fiehn, O., 2019. Systematic error removal using random forest for normalizing large-scale untargeted lipidomics data. *Analytical Chemistry*, 91(5), 3590–3596.

Pinu, F.R., Edwards, P.J., Jouanneau, S., Kilmartin, P.A., Gardner, R.C. and Villas-Boas, S.G., 2014. Sauvignon blanc metabolomics: Grape juice metabolites affecting the development of varietal thiols and other aroma compounds in wines. *Metabolomics*, 10, 556–573.

Smart, K.F., Aggio, R., Van Houtte, J.R. and Villas-Bôas, S.G., 2010. Analytical platform for metabolome analysis of microbial cells using methyl chloroformate derivatization followed by gas chromatography–mass spectrometry. *Nature Protocols*, 5(10), 1709–1729.

Villas-Bôas, S.G., Smart, K.F., Sivakumaran, S. and Lane, G.A., 2011. Alkylation or silylation for analysis of amino and non-amino organic acids by GC-MS? *Metabolites*, 1(1), 3–20.
