# Supplementary material for: Prediction of Feed Efficiency and Performance-Based Traits in Fish via Integration of Multiple Omics and Clinical Covariates
Source: Biology (Basel). 2023 Aug 15;12(8):1135. doi: 10.3390/biology12081135 (PMC10452023; doi:10.3390/biology12081135)
Supplement: Supplementary file 1 [file biology-12-01135-s001.zip › biology-2430619-supplementary/Methods S5 - General Composition.pdf]

Prediction of feed efficiency and growth traits in fish via integration of multiple omics and clinical covariates

Young et al. 2023

Supplementary Methods 5

1. General composition

1.1 Elemental analysis

Calcium and potassium in whole-body homogenates were determined using 2 g of sample via Inductively Coupled Plasma Mass Spectroscopy (ICP-MS [NexION® 300D]; Perkin Elmer Inc.: Waltham, MA, USA). Samples were accurately weighed into 50 mL Digi-tubes (SCP Science: Baie-D'Urfé, Quebec, Canada). Nitric acid (2.5 mL) and hydrochloric acid (0.5 mL) were added to each digestion vessel, which were heated for 60 mins at 85°C using a hot-block digestion system (SCP Science). Vessels were cooled, and Milli-Q water added to bring the total volume to 50 mL. Trace elements were determined on a 20 × dilution of the digest solution using ICP-MS. This instrument was configured with a Cetac ASX-520 auto-sampler (CETAC Technologies: Omaha, NE, USA), ESI FAST sample introduction valve, Peltier cooled PC3 spray-chamber, and a sea spray nebulizer. The instrument response was calibrated against certified standards, with the quantification using standard mode for Calcium at mass ^{43}Ca with a Rhodium internal standard and Kinetic Energy Discrimination mode for Potassium at mass ^{39}K with a Scandium internal standard. This protocol was developed and validated in-house and has been assessed and accredited under our laboratory's (Cawthron Institute) 17025 accreditation for metals in wide range of sample matrices.

1.2 Wet chemistry proximate analysis

Total protein was determined using the Kjeldahl technique according to method 981.10 ('Crude protein in meat. Block digestion method') of the AOAC International (AOAC 2019). Approximately 1 g of raw material was hydrolysed with 20 mL concentrated sulfuric acid (H_2SO_4) containing a Missouri Kjelgahl tablet (4.98 g K_2SO_4 and 0.02 g $CuCO_4.5H_2O$ [Buchi: Onelab, Auckland, NZ]) in a heat block (Buchi K446 [Buchi]). Sample digestion was made in 10 mL hydrogen peroxide (H_20_2). After digestion the solution was made alkaline, and the ammonia steam was distilled (Buchi K355 [Buchi]) into boric acid (H_3BO_3). The solution was titrated with 0.5N H_2SO_4. The amount of total nitrogen in the raw materials were multiplied by a conversion factor of 6.25 to determine total protein content per 100 g of sample.

Total lipid was determined according to method 948.15 ('Fat [crude] in seafood. Acid hydrolysis method') of the AOAC International (AOAC 2019). Approximately 8 g of sample was hydrolysed with 10 mL of hydrochloric acid in 2 mL ethanol and 4.4 mL H_2O on a stream bath for 15 min to free bound fat in cell membranes. The fat was extracted in Roese-Gottlieb extraction tubes from the aqueous acid solution with repeated 30 mL of diethyl ester and 25 mL of petroleum ether

extractions. Solvent was evaporated from the extract and the weight of residue expressed as total fat per 100 g of sample.

Moisture was measured by drying samples at 105°C to a constant weight according to a modified version of the official moisture in meat method (AOAC 950.46); modification from the referenced method is the raised temperature form 100–102°C to 105°C. Ash content was calculated accordance with the official method AOAC 920.153 by igniting 3–5 g of sample at 550°C to a constant weight in a muffle furnace. Carbohydrate content was determined by calculation: 100 – % crude protein – % total fat – % moisture – % ash.

1.3 Near infrared spectroscopy analysis

Based on our previous work (Miller et al. 2019), we also applied validated infrared spectroscopy models to reliably estimate total protein, lipid, moisture, and ash, and levels of fatty acid classes comprising saturated fatty acids (SFA), monounsaturated fatty acids (MUFA) polyunsaturated fatty acids (PUFA), and omega-3 fatty acids. Levels of eicosapentaenoic and docosahexaenoic acids were similarly estimated. Salmon homogenates were placed in a 50 mm rotating cup and scanned in reflectance mode using a Bruker MPA FT-NIRs (Bruker, Ettlingen, Germany). A background spectrum was generated every hour of use. Samples were scanned over the NIR region of 3,600–12,500 wavenumber (cm^{-1}). All scans were performed with an optical resolution of 16 cm^{-1} and a measurement time of 20.338 sec. OPUS 7.5 software (Bruker, Ettlingen, Germany) was used to analyse spectra. Interactive regions for each proximate (ash, lipid, moisture, protein, carbohydrates) were defined from wavenumber regions provided by Cozen (2003) for each analyte, and the fat absorption (C-H) vibration was used to model fatty acid components.

2. References

AOAC (Association of Official Agricultural Chemists), 2019. *International Official Methods of Analysis* (21st Edition). AOAC International, Maryland, USA. ISBN: 0-935584-89-7

Miller, M.R., Puddick, J., Symonds, J.E., Walker, S.P. and Tian, H., 2019. Application of a Fourier transform—near infrared reflectance spectroscopy method for the rapid proximate analysis of the greenshell mussel (*Perna canaliculus*) and king (Chinook) salmon (*Oncorhynchus tshawytscha*). *Aquaculture Research*, 50(6), 1668–1677.

Cozen, 2003. *Multivariate Calibration*. Ettlingen, Germany: Bruker Optik GmbH.
