# Supplementary material for: Prediction of Feed Efficiency and Performance-Based Traits in Fish via Integration of Multiple Omics and Clinical Covariates
Source: Biology (Basel). 2023 Aug 15;12(8):1135. doi: 10.3390/biology12081135 (PMC10452023; doi:10.3390/biology12081135)
Supplement: Supplementary file 1 [file biology-12-01135-s001.zip › biology-2430619-supplementary/Methods S6 - Fatty Acids.pdf]

Prediction of feed efficiency and growth traits in fish via integration of multiple omics and clinical covariates

Young et al. 2023

Supplementary Methods 6

1. Lipid fatty acid composition

Lipids were extracted for fatty acid analysis via a cold solvent modified Folch extraction (Folch et al., 1957). Samples (~5 g) were homogenised for 3 min in $CHCl_3$:MeOH:H_2O (40:40:36 final ratio) and then mixed for 10 min in a rotary wheel at 3000 rpm. After phase separation the organic phase was removed and concentrated prior to methylation via a saponification with methanolic sodium hydroxide. 0.2 g of extracted oil was refluxed in 4 mL of 0.5 M NaOH for 10 min prior to the addition of 5 ml Boron trifluoride (BF_3). After 5 min of further refluxing, 5 mL of heptane was added for 1 min reflux. Once cooled, 15 mL of saturated NaCl solution was added and thoroughly mixed. The heptane layer was collected and diluted prior to analysis for fatty acid methyl esters (FAMEs).

FAME samples were analysed according to AOAC official methods 963.22 ('Methyl esters of fatty acids in oils and fats. Gas chromatographic method' [AOAC 2019]) using an Agilent 6890 gas chromatography platform (Agilent Technologies; Victoria, Australia) fitted with an Agilent SP-2560 silica capillary column (100 m × 0.25 mm i.d., 0.2 µm film thickness [DKSH New Zealand Ltd; Auckland, New Zealand]) and flame ionised detector. Samples (1 µL) were injected via a split injector at 260°C. The column temperature was held at 220°C at 17 min, then raised by 2.8°C.min^{-1} to 240 °C and held for 5 min. Nitrogen was the carrier gas. Fatty acids were quantified by their peak areas and identified with an external commercial fatty acid standard (Supelco 37 Component FAME Mix [Merck; Auckland, New Zealand]) using ChemStation software (vA10.02) (Agilent Technologies; Auckland, New Zealand). Atotal of 32 FAMEs were quantified (C10:0 to C24:1).

2. References

Folch, J., Lees, M. and Sloane Stanley, G.H., 1957. A simple method for the isolation and purification of total lipids from animal tissues. *Journal of Biological Chemistry*, 226(1), 497–509.

AOAC (Association of Official Agricultural Chemists), 2019. *International Official Methods of Analysis* (21st Edition). AOAC International, Maryland, USA. ISBN: 0-935584-89-7
