# Supplementary material for: Prediction of Feed Efficiency and Performance-Based Traits in Fish via Integration of Multiple Omics and Clinical Covariates
Source: Biology (Basel). 2023 Aug 15;12(8):1135. doi: 10.3390/biology12081135 (PMC10452023; doi:10.3390/biology12081135)
Supplement: Supplementary file 1 [file biology-12-01135-s001.zip › biology-2430619-supplementary/Methods S7 - Blood Biomarkers.pdf]

Prediction of feed efficiency and growth traits in fish via integration of multiple omics and clinical covariates

Young et al. 2023

Supplementary Methods 7

1. Blood biomarkers

A selected suit of blood biomarkers with veterinary diagnostic relevance were assessed, as described in our previous work Casanovas et al. 2021. Blood samples were collected (without anti-coagulant) from the caudal vein immediately following euthanasia and aliquoted into four subsamples for further processing. Fresh peripheral blood samples were centrifuged (16,250 rcf; 8 min) to obtain plasma, transferred to 2 mL cryovials, snap frozen in liquid nitrogen, and stored at −80°C until analysis. Plasma samples (500 µL) were sent frozen on dry ice to an International Accreditation New Zealand (IANZ, https://www.ianz. govt.nz/) accredited commercial laboratory (Gribbles Veterinary; Christchurch, New Zealand) for a targeted and quantitative analysis of all biochemistry and haematology analyses as per International Federation of Clinical Chemistry and Laboratory Medicine (IFCC, htt ps://www.ifcc.org/) recommendations. Plasma samples were analysed for electrolytes and clinical chemistries using a Cobas c 501 automated chemistry analyser (Roche Diagnostics; Mannheim, Germany). Each of the assays used a standard kit developed for the autoanalyser. The analysis included total protein (gL^{-1}), albumin (gL^{-1}), alanine aminotransferase (IUL^{-1}), alkaline phosphatase (IUL^{-1}), aspartate aminotransferase (IUL^{-1}), glutamate dehydrogenase (IUL^{-1}), creatine phosphokinase (IUL^{-1}), creatinine ($\mu molL^{-1}$), calcium ($mmolL^{-1}$), chloride (mmol/L), cholesterol ($mmolL^{-1}$), glucose ($mmolL^{-1}$), magnesium ($mmolL^{-1}$), phosphate (mmol/L), potassium ($mmolL^{-1}$), sodium ($mmolL^{-1}$), triglycerides ($mmolL^{-1}$) and urea ($mmolL^{-1}$). Globulin (gL^{-1}) was calculated as the total protein minus albumin. Plasma cortisol (nmol/L) levels were determined using a Cobas e 411 automated endocrinology analyser (Roche Diagnostics). The assay used a standard kit developed for the autoanalyser (Roche Diagnostics, Mannheim, Germany). The inflammatory markers haptoglobin, C reactive protein (CRP), and prostaglandin E2 (PGE2) were analysed in plasma samples using ELISA kits (My BioSource; CA, USA) and read on a Spectramax® ABS plate reader (Molecular Devices; CA, USA).

2. References

Casanovas, P., Walker, S.P., Johnston, H., Johnston, C. and Symonds, J.E., 2021. Comparative assessment of blood biochemistry and haematology normal ranges between Chinook salmon (*Oncorhynchus tshawytscha*) from seawater and freshwater farms. *Aquaculture*, 537, 736464.
