# Supplementary material for: Prediction of Feed Efficiency and Performance-Based Traits in Fish via Integration of Multiple Omics and Clinical Covariates
Source: Biology (Basel). 2023 Aug 15;12(8):1135. doi: 10.3390/biology12081135 (PMC10452023; doi:10.3390/biology12081135)
Supplement: Supplementary file 1 [file biology-12-01135-s001.zip › biology-2430619-supplementary/Methods S8 - Haematology.pdf]

Prediction of feed efficiency and growth traits in fish via integration of multiple omics and clinical covariates

Young et al. 2023

Supplementary Methods 8

1. Haematology

Various haematological parameters were assessed, as described in our previous work Casanovas et al. 2021. Whole blood was used to prepare two blood smears per fish and air dried before sending to Gribbles Veterinary for staining (Leishman) and processing within 72 hrs of collection. White blood cell counts (WBC) were estimated based on the average counts of 10 fields. Data were presented as a range and absolute values of the differential were estimated based on the mean of this range. The differential leukocyte count was manually determined based on a count of 100 cells. An absolute value for lymphocytes, neutrophils and monocytes was determined from the fraction of the 100 cell differential multiplied by the mean of the WBC count range. For haemaglobin (Hb) analysis a 50–100 μL blood sample per fish was transferred to a 1.3 mL tube with lithium heparin and stored at 4°C until analysis, within 48 h of collection. Whole blood samples were analysed for total haemoglobin Hb content at Gribbles Veterinary Pathology (Christchurch, NZ) using a HemoCue® Hb201+ system (HemoCue®, Angelholm, Sweden). Heparinised micro haematocrit capillary tubes (Kimble Chase) were used for Haematocrit (Hct) determination. After sealing, the tubes were immediately centrifuged for 7 mins in a microhaematocrit centrifuge. Haematocrit and whole blood Hb content were used to calculate mean corpuscular haemoglobin concentration (MCHC) according to Houston (1997).

2. References

Casanovas, P., Walker, S.P., Johnston, H., Johnston, C. and Symonds, J.E., 2021. Comparative assessment of blood biochemistry and haematology normal ranges between Chinook salmon (*Oncorhynchus tshawytscha*) from seawater and freshwater farms. *Aquaculture*, 537, 736464.

Houston, A.H., 1997. Are the classical hematological variables acceptable indicators of fish health? *Transactions of the American Fisheries Society*, 126(6), 879–894.
